# Diabetic Foot Care Education and Practices: A Knowledge, Attitudes, and Practices Questionnaire-Based Study

**DOI:** 10.7759/cureus.85436

**Published:** 2025-06-05

**Authors:** Aishwarya Singh, Rohini R, Pankaj K Kannauje, Pranita Das, Jhasaketan Meher, Maninder Routray

**Affiliations:** 1 General Medicine, All India Institute of Medical Sciences, Raipur, Raipur, IND; 2 Medicine, All India Institute of Medical Sciences, Raipur, Raipur, IND

**Keywords:** diabetic foot disease, diabetic foot ulcer, kap for diabetic foot, michigan neuropath screening instrument, peripheral neuropathy

## Abstract

Background

Diabetic foot disease is a significant cause of morbidity among individuals with diabetes and can be effectively prevented with proper patient education early in the disease course. This study was conducted with the aim of assessing the knowledge, attitudes, and practices of diabetic patients toward foot care, as well as estimating the prevalence of diabetic foot disease.

Methods

A cross-sectional study was conducted among 128 diagnosed patients with diabetes, using a structured questionnaire and the Michigan Neuropathy Screening Instrument (MNSI) parts A and B, at a referral hospital in Central India. The questionnaire included questions about foot care practices, knowledge of diabetic foot complications, and attitudes toward foot care. The MNSI was used to screen for neuropathic symptoms and signs indicative of diabetic foot disease.

Results

Among the participants, 110 out of 128 (85.9%) reported that they had never received any education from their healthcare provider regarding proper foot care practices. Almost one-fourth of our participants, accounting for 95 (74.2%) of them, lacked adequate knowledge about maintaining proper foot hygiene, and 78 (60.9%) were unaware of the foot-related complications associated with diabetes. The study found a significant positive association between knowledge scores and the duration of diabetes. Despite the lack of knowledge, 125 (97%) of the patients demonstrated a positive attitude and expressed a willingness to adopt proper practices when educated. Notably, 52 (40.6%) of the participants walked barefoot, and 116 (90.6%) did not undergo annual foot examinations. Diabetic foot disease was identified in 40 out of 128 (31.3%) participants based on abnormal MNSI scores. There was a significant positive correlation between the duration of diabetes and the presence of diabetic foot disease. Higher MNSI score A was associated with poorly controlled diabetes, highlighting the impact of glycemic control on neuropathic symptoms. The presence of diabetic foot disease was also significantly associated with macroalbuminuria.

Conclusion

Our study revealed substantial deficiencies in the education of diabetic patients concerning foot care practices. The prevalent positive attitude among the patients indicates that diabetic foot disease can be effectively prevented with adequate patient education and proactive care by primary healthcare providers. Addressing these deficiencies in awareness and practice is essential for improving patient outcomes and preventing severe complications associated with diabetic foot disease.

## Introduction

Diabetes mellitus (DM), a significant chronic non-communicable disease, affects millions of individuals globally. The prevalence of diabetes is rapidly increasing, especially in newly industrialized and developing countries. As per the latest International Diabetes Federation (IDF) Diabetes Atlas data, 11.1% of the population worldwide is living with diabetes, with over 25% being unaware of their diagnosis. Of these, 81% of patients live in low- and middle-income countries. By 2030, the number of people with diabetes aged 20-79 will reach 642,800, with projections of around 783,700 by 2045 [1].

The term “diabetic foot” covers diabetic peripheral neuropathy, peripheral vascular disease, Charcot neuroarthropathy, foot ulcers, and osteomyelitis, potentially leading to amputation [2]. Neuropathy in diabetes may take various forms, such as distal symmetric polyneuropathy and autonomic neuropathy, impacting cardiovascular, gastrointestinal, urogenital, and sudomotor functions [3]. Understanding these complications is crucial for improving diabetic foot care and managing diabetes-related health issues effectively.

Such complications are exacerbated by infections, which can result in ulcer formation. Foot ulcers affect approximately 19-34% of diabetic individuals over their lifetime, severely impacting their quality of life through morbidity, reduced mobility, loss of work, diminished income, and decreased social engagement [4]. Individuals with diabetes face a 25-fold increased risk of lower limb amputation compared to those without diabetes. Zhang et al. estimated that diabetic foot complications alone resulted in 16.8 million years lived with disability (YLDs) (2.07% of global YLDs) in 2016, which included 12.9 million YLDs from neuropathy only, 2.5 million YLDs from foot ulcers, 1.1 million YLDs from amputation with no prosthesis, and 0.4 million YLDs from amputation with prosthesis [5]. Effective control over blood glucose levels, blood pressure, and cholesterol can delay or even prevent these diabetes-related complications [6].

Preventing and managing diabetic foot disease (DFD) requires a comprehensive strategy that includes screening, patient education, proper footwear, and management of risk factors.

However, studies reveal a significant lack of awareness about diabetic foot conditions among patients and primary caregivers who often do not perform routine foot examinations during hospital visits [7-9]. Addressing these deficiencies in awareness and practice is essential for improving patient outcomes and preventing severe complications.

This study was conducted with the aim of assessing the current knowledge, attitudes, and practices (KAP) of diabetic patients towards diabetic foot care and estimating the prevalence of DFD. We meant to understand the importance of early detection and proactive care by primary healthcare providers. We also intended to fill in the gap of meager studies conducted in this regard in our country, and especially in our region.

Aim and objectives

The primary objective of this study was to assess the knowledge, attitudes, and practices regarding foot care in patients with DM in Central India. The secondary objectives included studying the clinical profile of DFD and association of DFD with HbA1c.

## Materials and methods

We conducted the study among patients with DM at the Outpatient Department of General Medicine at a referral center in Central India after approval from the All India Institute of Medical Sciences (AIIMS) Raipur Institute Ethics Committee with approval number AIIMSRPR/IEC/2022/1254. 

Study design

This was a cross-sectional, descriptive, questionnaire-based observational study.

Study setting

This was conducted in the Outpatient Department of General Medicine at AIIMS, Raipur (Chhattisgarh).

Study population

The study population included patients diagnosed with DM in the Outpatient Department of General Medicine at AIIMS, Raipur (Chhattisgarh).

Inclusion criteria

Participants were eligible for inclusion if they met all of the following criteria: a) age ≥ 18 years, b) diagnosed case of DM, and c) provided informed consent to participate in the study.

Exclusion criteria

Participants were excluded from the study if they had any of the following conditions: a) chronic kidney disease (Kidney Disease Improving Global Outcomes (KDIGO) stage 3 or more), b) chronic liver disease, c) diagnosed systemic malignancy, d) uncorrected vitamin B12 deficiency, and e) features suggestive of etiologies other than diabetic neuropathy.

Sample size

It was calculated based on previous articles [10] in which the prevalence of DM was 9%. The sample size was calculated using the formula:

n = (Z^2^. p. q)/e^2^

where

n = desired sample size; Z = 1.96 (A point on normal distribution with 95% confidence level) p = 9% (0.09) (prevalence of DM); q = 1-p = 100%-9% = (0.91); e = 0.05 (desired precision); and n (sample size) = (1.96)^2^ (0.09)(0.91) / (0.05)^2^ = 125.88, rounding off to 126.

Sampling technique

The sampling technique used for this study was consecutive sampling.

Study duration

The study was conducted over a period of 18 months, from November 2022 to April 2024.

Study tools

a) The KAP questionnaire was translated back and forth in the local language and validated by doing a pilot study on 30 participants. The tables in the later sections show the questionnaires for KAP, respectively (Appendix A). b) DFD was assessed using the standard Michigan Neuropathy Screening Instrument (MNSI) parts A and B (Appendix B) [11]. While MNSI part A is a self-administered questionnaire that assesses the symptoms of the patients, MNSI part B is administered by the clinician and assesses the various signs of peripheral neuropathy, peripheral vascular disease, foot ulcers, and various other deformities. The MNSI is developed by the Michigan diabetes research center and is free to download from their website [11]. The project described was supported by grant number P30DK020572 (MDRC) from the National Institute of Diabetes and Digestive and Kidney Diseases. Part A of the MNSI questionnaire was translated back and forth in the local language (Hindi), and a pilot study was done to validate the same. 

All eligible patients, after obtaining informed consent, were administered the MNSI part A questionnaire, followed by the KAP questionnaire. Finally, the MNSI part B examination was done, and the investigation reports of the patients were noted.

The data were entered into Microsoft Excel (Microsoft® Corp., Redmond, WA) and cleaned. The cleaned data were analyzed using SPSS (IBM SPSS Statistics for Windows, IBM Corp., Version 25, Armonk, NY). The statistical analysis comprised calculating the mean, median, and proportions. Chi-square test (Fisher’s exact test wherever applicable) was used for comparing the proportion between the two groups. The Shapiro-Wilk test was used to assess the normality of the data. For normally distributed data, the t-test was used to compare the means between the two groups. For skewed distribution, the Mann-Whitney U test was used to compare the median between the two groups, and Spearman’s rho was used to calculate the correlation coefficient. The level of significance was taken as P < 0.05.

## Results

We included 128 diabetic patients in our study. The baseline demographic characteristics of the study population are summarized in Table 1. The metabolic profile of the study population is given in Table 2. The mean age of the population was 54.6 (±11) (95% CI, 52.7-56.5) years. The mean BMI was 21 (8 (±6.3) (95% CI, 20.7-22.9) kg/m²). A total of 117 (91.4%) of our study participants had a normal Ankle Brachial Pressure Index (ABPI). The mean HbA1c was 8.8%(±0.99%) (95% CI, 8.6-8.97), with only 41 (32%) of the subjects having good control as defined by a HbA1c of ≤7%. A low-density lipoprotein (LDL) target of less than 100 mg/dL was met in 78 (60.9%) of patients.

**Table 1:** Baseline demographic characteristics of study population

Variables	n (%)
Age group (in years)	
<40	13 (10.2%)
41-60	74 (57.8%)
>60	41 (32 %)
Gender	
Female	46 (35.9%)
Male	82 (64.1%)
Residence	
Urban	115 (89.8%)
Rural	13 (10.2%)
Education	
Illiterate	12 (9.4%)
Primary school	34 (26.6%)
Secondary school	42 (32.8%)
Graduate	21 (16.4%)
Postgraduate	19 (14.8%)
Occupation	
Unemployed	42 (32.8%)
Semiskilled	42 (32.8%)
Skilled	20 (15.6%)
Professional	18 (14.1%)
Self employed	6 (4.7%)
Socio-economic class	
Upper	6 (4.7%)
Upper-middle	29 (22.7%)
Lower-middle	55 (43%)
Upper-lower	38 (29.7%)
Lower-lower	0
Duration of diabetes (in years)	
<5	46 (35.9%)
5-10	33 (25.8%)
>10	49 (38.3%)
Types of diabetes	
Type 1	5 (3.9%)
Type 2	98 (76.6%)
Chronic pancreatitis-associated diabetes mellitus	5 (3.9%)
Others	20 (15.6%)
Insulin use	
Yes	22 (17.2%)
No	106 (82.8%)

**Table 2:** Metabolic profile of study population MNSI - Michigan Neuropathy Screening Instrument The WHO Asian classification of BMI has been used.

Variable	n (%)
Body mass index (kg/m^2^)	
Underweight (<18.5)	6 (4.7%)
Normal (18.5-22.99)	36 (28.1%)
Overweight (23-24.99)	22 (17.2%)
Obesity class- I (25-29.99)	43 (33.6%)
Obesity class-II (30 and above)	21 (16.4%)
HbA1c (%)	
Good control (<7%)	41 (32%)
Poor control (>7%)	87 (68%)
Urine-albumin creatinine ratio (mg/g)	
Normal (<30)	75 (58.6%)
Microalbuminuria(30-300)	43 (33.6%)
Macroalbuminuria (>300)	10 (7.8%)
MNSI A	
Normal (score <7)	112 (87.5%)
Abnormal (score≥7)	16 (12.5%)
MNSI B	
Normal (score <2.5)	90 (70.3%)
Abnormal (score ≥2.5)	38 (29.7%)

The KAP questionnaire findings are detailed in the tables below. It was observed that patients had limited knowledge about foot care (Table 3). The mean knowledge score was 3.27 (95% CI, 2.71,3.79) out of a total of 10. Among the study population, 78 patients (60.9%) were unaware that diabetes increases the risk of foot problems, and 66 (51.6%) lacked knowledge regarding the heightened risk of peripheral neuropathy; 98 (76.6%) were unaware of increased risk of peripheral vascular disease both of which increase the risk of diabetic foot ulcers. Furthermore, 95 (74.2%) of patients did not know how to maintain proper foot hygiene. As shown in Table 3, very few knew how to cut nails properly, or regarding proper footwear. The dismal part was that 110 patients (85.9%) had never received any proper education regarding foot care from their primary physicians. There was a significant association between higher knowledge scores and longer diabetes duration (P < 0.05) in our study. We also found a significant association between median knowledge score and educational status (P < 0.05).

**Table 3:** Knowledge questionnaire and frequency

Knowledge questions	n (%)
1. Do you know that having diabetes increases the risk of foot problems?	
Yes	50 (39.1%)
No	78 (60.9%)
2. Do you know diabetics are likely to develop reduced sensation in their feet and are more prone to have foot ulcers?	
Yes	62 (48.4%)
No	66 (51.6%)
3. Do you know that cracked heels can lead to foot infection?	
Yes	55 (43%)
No	73 (57%)
4. Do you know it is important to inspect your feet regularly?	
Yes	52 (40.6%)
No	76 (59.4%)
5. Do you know it is important to examine the inside of footwear for any objects or tears?	
Yes	58 (45.3%)
No	70 (54.7%)
6. Do you know that poor circulation in the feet may result from smoking, and then you are more prone to get foot ulcers?	
Yes	30 (23.4%)
No	98 (76.6%)
7. Do you know how to properly maintain the hygiene of your feet?	
Yes	33 (25.8%)
No	95 (74.2%)
8. Do you know that there is a specific kind of footwear you should wear?	
Yes	29 (22.7%)
No	99 (77.3%)
9. Do you know how to properly cut your nails?	
Yes	39 (30.5%)
No	89 (69.5%)
10. Were you given any information regarding foot care?	
Yes	18 (14.1%)
No	110 (85.9%)

On the contrary, most patients had the attitude to adopt good practices when given proper education and advice (Table 4). The mean attitude score was 3.90 out of 4.

**Table 4:** Attitude questionnaire and frequency

Attitude questions	n (%)
1. Are you willing to perform regular exercise and change your food habits to prevent further diabetic complications?	
Yes	126 (98.4%)
No	2 (1.6%)
2. Are you willing to take the responsibility of daily examination of your feet and footwear, as well as regular foot‐care specialist consultation?	
Yes	125 (97.7%)
No	3 (2.3%)
3. Are you willing to use special footwear advised by the foot care specialist?	
Yes	123 (96.1%)
No	5 (3.9%)
4. Do you think people with diabetes should take the responsibility of self-foot care?	
Yes	125 (97.7%)
No	3 (2.3%)

The study participants fared better as far as practices were concerned (Table 5). The mean practice score was 5.71 (95% CI, 5.39,6.03) out of 10. As expected culturally in our setup, almost 52 (40.6%) of the participants walked barefoot, at least indoors. A total of 67 (52%) of the participants inspected their feet regularly for injury; 116 (90.6%) of the participants cut their nails using proper instruments, 93 (72.7%) washed their feet regularly, and 91 (71.1%) examined their shoes before wearing them and were willing to consult a podiatrist for foot related issues. However, 116 (90.6%) of the participants did not go for annual foot check-ups as recommended by the American Diabetes Association (ADA).

**Table 5:** Practice questionnaire and frequency

Practice questions	n (%)
1. Do you walk barefoot?	
Yes	52 (40.6%)
No	76 (59.4%)
2. Do you examine your feet for any marks/injury?	
Yes	67 (52.3%)
No	61 (47.7%)
3. What would you do if you find any foot abnormality?	
Manage yourself	37 (28.9%)
Consult a podiatrist	91 (71.1%)
4. Do you wash your feet daily and carefully dry the cleft between toes after washing?	
Yes	93 (72.7%)
No	35 (27.3%)
5. Are your toe nails cut straight through regularly?	
Yes	100 (78.1%)
No	28 (21.9%)
6. What type of cutting instrument do you use?	
Pointed tip cutter/scissors	12 (9.4%)
Rounded-tip scissors/nail cutter	116 (90.6%)
7. Do you examine your shoes before wearing them?	
Yes	91 (71.1%)
No	37 (28.9%)
8. Do you use comfortable, closed, and soft footwear?	
Yes	112 (87.5%)
No	16 (12.5%)
9. Do you change your footwear regularly?	
Yes	64 (50%)
No	64 (50%)
10. Do you go for foot check-up regularly?	
Yes	12 (9.4%)
No	116 (90.6%)

We found a weak but significant positive correlation between knowledge and practice scores (r = 0.22, P < 0.05). There was no association between the educational status and mean attitude and mean practice score; socioeconomic status and mean KAP score; duration of diabetes and mean KAP score. There was no significant difference between males and females in the mean KAP score.

Our secondary objective included the study of the clinical profile of DFD. We defined DFD as an abnormal MNSI part A (a score of ≥7) and/or part B (a score of ≥2.5). Forty of 128 (31.3%) participants had DFD. Only 16 patients had an abnormal MNSI part A score, whereas MNSI part B was abnormal in 38 patients. Twelve participants among the 40 (30%), had foot deformity, six (15%) had foot ulcers, vibration perception threshold (VPT) was reduced in 25(62.5%), 10-g monofilament perception was absent in 20 (50%), ankle reflex was absent in 27 (67.5%). Among the patients with foot deformities, seven (58.3%) had claw feet, and five (41%) had Charcot foot. Among patients with Charcot foot, four (80%) had mid-foot involvement, followed by one (20%) with forefoot involvement. Two among these had associated DFU.

The majority of individuals with DFD in our study had a duration of diabetes greater than 10 years (55.5%). Patients with a duration of diabetes greater than 10 years (Table 6) had proportionally higher rates of foot deformities, foot ulcers, absent ankle reflexes, diminished VPT, and absent monofilament perception. However, it reached statistical significance only in the absence of ankle reflexes and absence of 10-g monofilament perception.

**Table 6:** Association of diabetic foot disease with duration of diabetes P-value was calculated using the chi-square test. P < 0.05 is considered significant, marked with *. Values in brackets are percentages of "n" of respective columns.

Variables	Duration of diabetes		P-value
	<10 years, n = 79	>10 years, n = 49	
Any deformity			
Present	08 (10.1%)	10 (20.4%)	0.10
Absent	71 (89.9%)	39 (79.6%)	
Foot ulceration			
Present	03 (3.8%)	04 (8.2%)	0.29
Absent	76 (96.2%)	45 (91.8%)	
Ankle reflex			
Present	59 (74.7%)	27 (55.1%)	0.02*
Absent	20 (25.3%)	22 (44.9%)	
Vibration perception			
Present	52 (65.8%)	27 (55.1%)	0.22
Absent	27 (34.2%)	22 (44.9%)	
Monofilament perception			
Present	68 (86.1%)	32 (65.3%)	0.006*
Absent	11 (13.9%)	17 (34.7%)	

Regarding ankle reflexes, 42 participants (32.8%) had absent reflexes. Among those with absent reflexes, 27 (64.3%) had a BMI over 23 kg/m². In those with DFD, 67.5% had absent ankle reflexes. Our study confirmed a significant correlation between absent ankle reflexes and foot deformities or ulcers (P < 0.05).

In our study, 28 participants (21.9%) showed loss of monofilament perception, primarily among those aged 41-60 years and predominantly in the urban population. Additionally, 16 participants (57.1%) with lost monofilament perception had a BMI greater than 23 kg/m². We did not find a statistically significant link between loss of monofilament perception and foot deformities (P = 0.22). However, there was a significant association between loss of monofilament perception and DFUs (P < 0.05)

Among the total study population, a BMI greater than 23 kg/m² was present in 86 (67.2%), which makes them overweight or obese as per Asian standards. Table 7 shows the breakdown of DFD among this subgroup of our population. We noted that deformities were more common in individuals with higher BMIs. However, none of the associations showed statistical significance.

**Table 7:** Association of diabetic foot disease with body mass index P-value calculated using the chi-square test, for all except *, where the Fisher exact test was used. Values in brackets indicate the percentage of “n” of the respective column.

Variables	Body mass index		P-value
	<23 kg/m^2^, n = 42	≥23 kg/m^2^, n = 86	
Any deformity			
Present	5 (11.9%)	13 (15.1%)	0.62
Absent	37 (88.1%)	73 (84.9%)	
Diabetic foot ulcer			
Present	4 (9.5%)	3 (3.5%)	0.22*
Absent	38 (90.5%)	83 (96.5%)	
Ankle reflexes			
Present	27 (64.3%)	59 (68.6%)	0.63
Absent	15 (35.7%)	27 (31.4%)	
Vibration perception			
Present	22 (52.4%)	57 (66.3%)	0.13
Absent	20 (47.6%)	29 (33.7%)	
10-g monofilament perception			
Present	30 (71.4%)	70 (81.4%)	0.20
Absent	12 (28.6%)	16 (18.6%)	

We observed a significant association between MNSI A scores and poorly controlled diabetic status (HbA1c > 7%), as shown in Table 8.

**Table 8:** Association of MNSI A and HbA1c among study participants MNSI, Michigan Neuropathy Screening Instrument P < 0.05 showing significance. Calculated using Fisher’s exact test.

HbA1c	MNSI A		P-value
	Abnormal n (%)	Normal n (%)	
Bad control (>7%)	15 (17.2%)	72 (82.8%)	0.02
Good control (<7%)	1 (2.4%)	40 (97.6%)	

**Figure 1:**
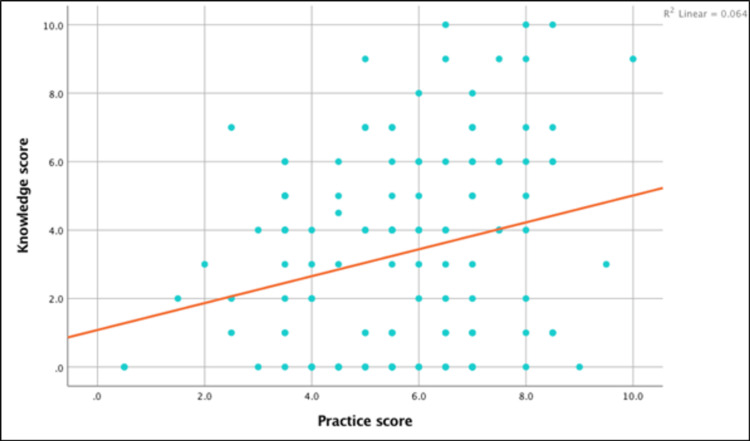
Scatter plot showing correlation between MNSI A and MNSI B, correlation coefficient 0.47 (P < 0.01) MNSI - Michigan Neuropathy Screening Instrument

We found a positive correlation between scores of MNSI parts A and B. Though predictable and significant, the correlation was not very strong (r = 0.47, P < 0.05), as shown in Figure 1.

DFUs were more common in participants with absent ankle jerks as well as those with absent 10-g monofilament perception (Table 9 and Table 10, respectively).

**Table 9:** Association between diabetic foot ulcer and ankle reflexes P < 0.05 showing significance. Calculated using Fisher’s exact test.

Diabetic foot ulcer	Ankle reflex		P-value
	Present n (%)	Absent n (%)	
Absent	84 (97.7%)	37 (88.1%)	0.04
Present	2 (2.3%)	5 (11.9%)	

**Table 10:** Association between diabetic foot ulcer and 10-g monofilament perception P < 0.05 showing significance. Calculated using Fisher’s exact test.

Diabetic foot ulcer	10-g monofilament perception		P-value
	Present (%)	Absent (%)	
Absent	97 (97.0%)	24 (85.7%)	0.04
Present	3 (3.0%)	4 (14.3%)	

Among our study participants, 53 (41.4%) had nephropathy, including 43 (33.6%) with microalbuminuria and 10 (7.8%) with macroalbuminuria (Table 1). Of those with DFD, 20 individuals (50%) had nephropathy (Table 11). A significant association was found between urine albumin-creatinine ratio and DFD, with a notably higher proportion of macroalbuminuria among those with DFD (P = 0.02).

**Table 11:** Association between diabetic foot disease and nephropathy P < 0.05 showing significance. Calculated using the chi-square test.

Urine albumin creatinine ratio (mg/g)	Diabetic foot disease		P-value
	Present, n (%)	Absent, n (%)	
Normal (<30)	20 (50.0%)	55 (62.5%)	0.02
Microalbuminuria (30-300)	13 (31.6%)	30 (34.1%)	
Macroalbuminuria (>300)	7 (18.4%)	3 (3.4%)	

## Discussion

This study evaluated the KAP related to diabetic foot care among patients, along with an assessment of peripheral neuropathy using standardized tools. The findings were compared with multiple national and international studies, highlighting significant regional and contextual differences in diabetic foot care awareness and management.

With regard to the knowledge of DFD, our study found that only 14.1% of the population had received some education about foot care. This is similar to a study conducted in North India, where only 12.5% of individuals attending a tertiary care hospital had previously received foot care advice from healthcare professionals [12]. However, research from South India presents a different perspective, revealing that approximately 75% of participants had good knowledge scores, and 67% demonstrated good foot care practices [13]. Similarly, the results of a study by Maha Obaid Alharbi et al, where 56.9% of participants had received knowledge about diabetic foot care in the past, were substantially higher compared to our study [2]. This variation underscores the differences in foot care education and practices across different regions. We also found a significant association between median knowledge score and the educational status of the participants, similar to a study conducted by Karthik et al. [9]. This can be expected as basic education does increase the understanding of the disease process. However, contrary to the same study, we did not find any association with the occupation of the participants.

We highlight a crucial gap in patient education within our study population. The lack of formal education on diabetic foot care in our cohort may be attributed to numerous factors such as limited access to healthcare services, insufficient healthcare infrastructure, and the potential lack of trained healthcare professionals who can provide the necessary education.

Furthermore, while a study by Al Amri et al. reported high awareness levels (e.g., 66.7% knew about poor blood flow to diabetic feet, and 51.4% knew about the impact of smoking), our study found only 48.4% understood the risk of reduced sensation, and just 23.4% knew smoking negatively impacts foot circulation [14]. These gaps reinforce the urgent need for targeted educational programs.

However, despite the low knowledge levels, 97.7% of participants in our study expressed a positive attitude toward diabetic foot care, including the willingness to conduct daily self-examinations and visit specialists regularly. This favorable attitude suggests that given appropriate education, patients are open to adopting preventive behaviors.

This finding aligns with studies from Ethiopia and Benin. In Ethiopia, 65.2% of participants with good attitudes were also from urban and better-educated backgrounds [15]. Similarly, the Benin study found that while attitudes were generally positive, there were gaps in translating these attitudes into consistent practices [16]. Our results suggest that education could bridge this gap, enabling patients to transform awareness into sustainable behavioral changes.

Despite the positive attitudes, practices varied. In our cohort, 52.3% of participants examined their feet regularly, closely mirroring results from a study by Hanif et al. (52%) [17]. Foot washing was more commonly practiced, with 93 (72.7%) washing and drying between the toes, a practice possibly driven by cultural norms in India. 

We found no association between occupation, socio-economic class, and the KAP score of our participants. A similar study conducted in a southern state of India [9], however, did show a positive association between the same. The possible reason why we did not find an association could be because of the small sample size and uneven distribution of our participants among subgroups.

Interestingly, 100 (78.1%) of participants correctly trimmed their nails straight, significantly higher than the 36% reported in the comparative study. Barefoot walking, a risky behavior, was observed in 52 (40.6%) of participants, lower than the 45% found in the reference study [17]. Moreover, 91 (71.1%) of our participants checked the inside of their shoes, much higher than the 42.66% observed in the reference group. These differences likely reflect variations in literacy, healthcare education, and cultural habits.

However, 90.6% of participants in our study did not undergo regular foot check-ups, indicating a gap in preventive healthcare utilization. Still, most said they would seek professional advice upon noticing injuries, revealing a reactive rather than proactive approach to foot care.

In a cross-sectional study by Al Amri et al., 47% of participants reported that they only change their shoes when they are damaged, while 35.9% changed their shoes more than once a year [14]. The same study also found that 70.4% of patients only consulted a physician if they observed deformities such as wounds or ulcers in their feet. In contrast, our study shows that a staggering 116 participants (90.6%) do not engage in regular foot checkups. However, 91 participants (71.1%) indicated that they would seek advice from a foot specialist if they noticed any marks or injuries during a foot examination. This disparity underscores differences in the level of counselling and education provided by healthcare professionals regarding the importance of recognizing and addressing symptoms of DFD.

A significant positive correlation was found between knowledge and practice, reinforcing similar findings by Giruzzi and Pourkazemi et al. [18,19]. Their studies noted that increased knowledge scores correlated with improved foot care behaviors and attitudes. This emphasizes the need for structured educational initiatives to ensure knowledge is effectively translated into practice.

Interestingly, while knowledge increased with the duration of diabetes (>10 years), there was no significant difference in attitude and practice scores across groups. This suggests that without continuous intervention, time alone does not improve behavior.

Our study revealed a statistically significant positive correlation between MNSI A and MNSI B. This finding aligns with existing literature, suggesting that these assessment tools are reliable in evaluating neuropathy. However, we did find that a greater number of our patients had an abnormal MNSI part B score compared to an abnormal MNSI part A score. Sutkowska et al. demonstrated that part B of the MNSI is stronger in detecting neuropathy because it is more objective than part A, which relies more on patient-reported symptoms [20]. This objectivity is crucial in clinical settings where subjective biases can affect the accuracy of neuropathy detection. This signifies that even patients who are asymptomatic for any symptoms of diabetic neuropathy may have neuropathy on examination. Thus, it is important to examine the feet of the patients even if they have no symptoms.

Additionally, we observed a significant association between MNSI A and poorly controlled diabetic status (HbA1c > 7%). Specifically, patients with higher HbA1c levels had significantly higher abnormal MNSI A scores than those with better glycemic control (P < 0.05), which is in line with the established fact that diabetic neuropathy complicates uncontrolled diabetes.

Several studies have focused on the association between neuropathy and nephropathy in diabetic patients. Nabrdalik et al. demonstrated that diabetic kidney disease was present in 18 (26%) patients with diabetic peripheral neuropathy and only four (3.5%) patients without neuropathy [21]. Our study further reinforced this finding, confirming a positive association between neuropathy and proteinuria.

Patients with a duration of diabetes greater than 10 years had significantly higher rates of foot deformities, foot ulcers, absent ankle reflexes, diminished VPT, and impaired monofilament perception in our study. The majority of individuals with DFD had a duration of diabetes greater than 10 years (55.5%). This finding is consistent with a cross-sectional multicentric study in the United Kingdom by Young et al., which found that the prevalence of peripheral neuropathy increased with the duration of diabetes [22]. The study reported neuropathy prevalence rates of 20.8% for those with diabetes duration less than five years, rising to 36.8% for those with a duration greater than 10 years. Piran et al. identified a significant link between the duration of diabetes and the risk of developing diabetic foot ulcers [23]. Their research showed that 56.3% of patients with diabetes for more than 10 years had diabetic foot ulcers, whereas 28.7% did not show signs of neuropathy. Our findings are consistent with this, as 55% of patients with DFD had diabetes for over a decade, compared to 30.7% of those without DFD. This resulted in a statistically significant correlation between the duration of diabetes and the presence of DFD (P < 0.01).

Higher BMI in our study was associated with increased risk of neuropathy and deformities. Increased weight puts more pressure on the feet, leading to a greater risk of foot deformities and ulcers, which are critical factors in the development of DFD.

In our study, 28 participants (21.9%) showed loss of monofilament perception. Although our research showed a significant association between monofilament loss and the presence of foot ulcers (P = 0.04), indicating that monofilament testing is more effective for detecting established damage rather than early disease, it should not be solely relied upon for diagnosing at-risk diabetic feet. According to a study by Dros et al., the sensitivity of monofilament testing ranged from 41% to 93%, while specificity varied between 68% and 100% [24]. These discrepancies may stem from differences in how the test is performed, the sites of application, and interpretation by different observers.

Regarding ankle reflexes, 42 participants (32.8%) had absent reflexes. A study by Shehab et al. highlighted the ankle reflex as having high sensitivity (91.5%) and moderate specificity (67.4%) for detecting diabetic peripheral neuropathy, suggesting it is an effective bedside tool when used alongside vibration perception testing (VPT) [25]. Our study confirmed a significant correlation between absent ankle reflexes and foot deformities or ulcers. Another study where the association of ankle reflex was seen with VPT itself by Malik et al. found a significant agreement between VPT and ankle reflex with κ = 0.538 (P < 0.0001), concluding sensitivity and specificity of ankle reflex of 81.09 and 81.67% respectively, with diagnostic accuracy of 81.22% [26]. Given its ease of use and straightforward interpretation, ankle reflex testing can be a practical alternative to VPT for early detection of diabetic foot issues, potentially preventing further complications.

A majority (87, 68%) of our population had poor control of HbA1c. Among these individuals, 30 (34.48%) had DFD, while among the patients with good control of HbA1c, only 10 (24.39%) had DFD. Despite these clear numerical differences, statistical analysis revealed no significant association between DFD and HbA1c levels (P = 0.25). This lack of statistical significance may be influenced by factors such as the study's sample size or the duration over which HbA1c levels were monitored.

A cross-sectional study by Su et al. demonstrated that 18.1% of patients with diabetic peripheral neuropathy had higher HbA1c levels than patients with good control of HbA1c [27]. This study supports the notion that poor glycemic control is associated with an increased risk of neuropathy. However, the precise relationship between HbA1c levels and the development of DFD may involve more complex mechanisms and risk factors beyond glycemic control alone.

Beyond neuropathy, our study revealed a significant association between nephropathy and HbA1c. Specifically, the proportion of individuals with nephropathy was significantly higher among those with poor HbA1c control (HbA1c > 7%) compared to those with normal HbA1c levels (P = 0.02). This finding is in line with research from other studies, which have consistently shown that higher HbA1c levels are correlated with an increased risk of nephropathy and other microvascular complications.

The strengths of our study are that we included a comprehensive KAP questionnaire, which was meticulously administered. We also used an established MNSI questionnaire to diagnose DFD. We did multiple subgroup analyses to try to find our various associations and correlations, and found some important results.

We had some limitations with our study. With only 128 participants and conducted in a single center, the sample size may be too small to represent the full diversity of DFD across a larger population, potentially affecting the reliability and generalizability of the results. Being a cross-sectional study, it limits the ability to determine causality or observe changes over time, which is important for understanding the progression of DFD. Also, there was a selection bias in the study, as ours is a referral center.

Based on the findings from the study on DFD and patient awareness, future longitudinal research can be undertaken to study the progression of DFD and the effect of diabetes foot care education on the improvement of foot care practices among patients. Research can also be undertaken to assess the sensitivity of various diagnostic tests for the assessment of diabetic neuropathy.

## Conclusions

The findings underscore the need for proactive, educational, and clinical interventions in diabetic foot care, particularly in regions with limited healthcare access. Improving patient knowledge and reinforcing positive practices through regular follow-ups can reduce the burden of DFD and its complications, improving both quality of life and healthcare outcomes. These changes ought to come from the primary care level at our setup, where the patient first presents usually for several years, before presenting to higher centers for diabetes management. The primary health care providers need to be sensitized regarding the need for educating the diabetic patient at the time of diagnosis, the need for regular and proper foot care, and then reinforcing this at subsequent visits. Only then will we be able to prevent the morbidity and mortality associated with DFD.

## Figures and Tables

**Figure 2:**
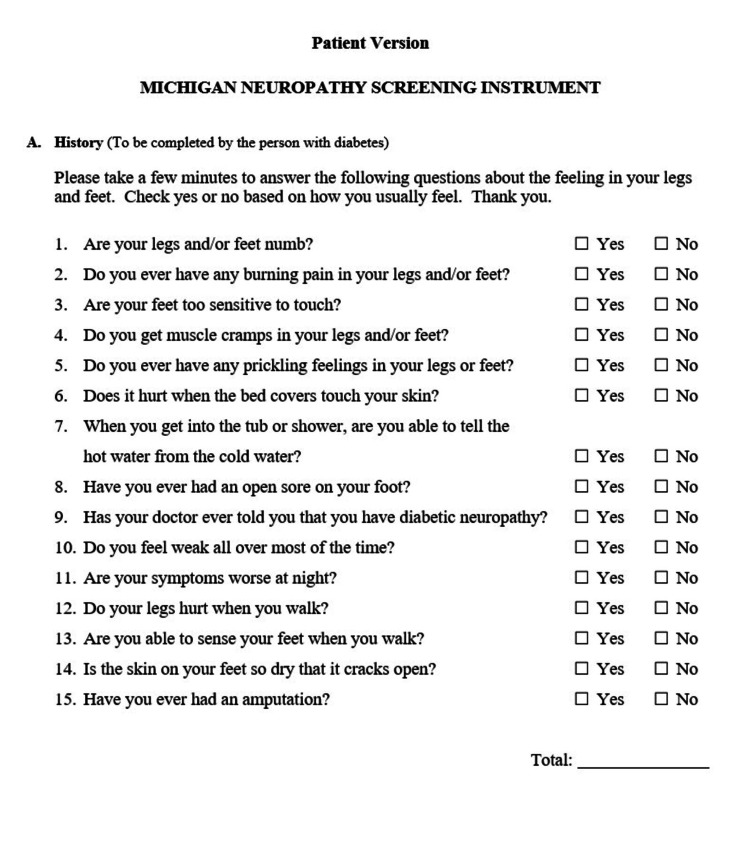
Michigan Neuropathy Screening Instrument Part A Downloaded from [11]

**Figure 3:**
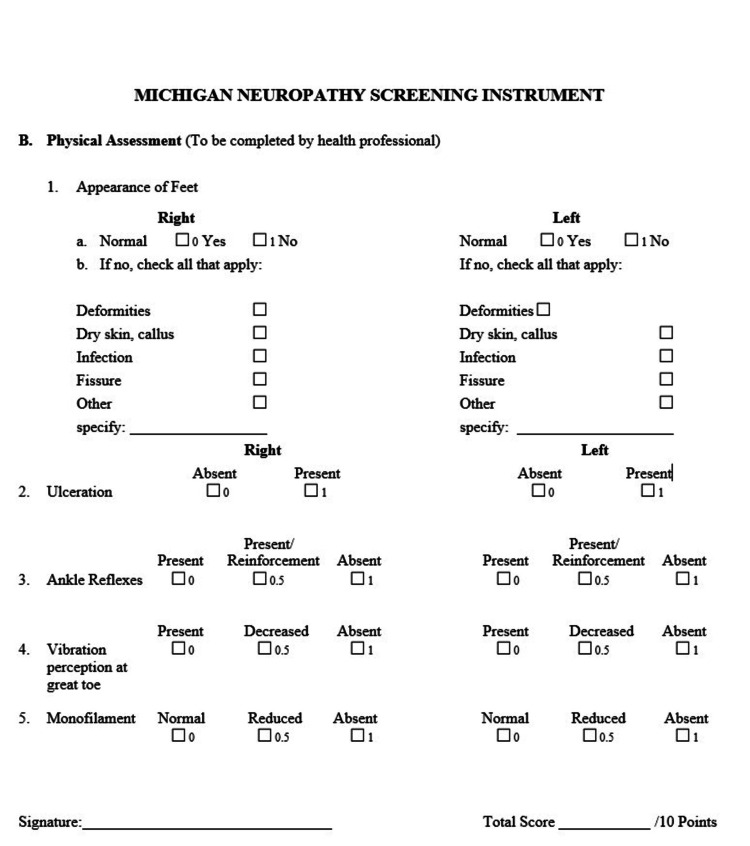
Michigan Neuropathy Screening Instrument Part B Downloaded from [11]

## Appendices

Appendix A

Knowledge, Attitudes, and Practices (KAP) Questionnaire

**Table 12:** Attitude questionnaire

S. no.	Questions	Options		Scoring (by the investigator)
1.	Are you willing to perform regular exercise and change your food habits to prevent further diabetic complications?	Yes	No	
2.	Are you willing to take the responsibility of daily examination of your feet and footwear, as well as regular footcare specialist consultation?	Yes	No	
3.	Are you willing to use special footwear advised by the foot care specialist?	Yes	No	
4.	Do you think people with diabetes should take the responsibility of self-foot care?	Yes	No	
Total score				

**Table 13:** Practice questionnaire

S. no.	Questions	Options		Scoring (by the investigator)
1.	Do you walk barefoot?	Yes	No	
2.	Do you examine your feet for any marks/injuries?	Yes	No	
3.	What would you do if you found any foot abnormality?	Manage yourself	Consult a podiatrist	
4.	Do you wash your feet daily and carefully dry the cleft between your toes after washing?	Yes	No	
5.	Are your toenails cut straight through regularly?	Yes	No	
6.	What type of cutting instrument do you use?	Pointed tip cutter/scissors	Rounded-tip scissors/nail cutter	
7.	Do you examine your shoes before wearing them?	Yes	No	
8.	Do you use comfortable, closed, and soft footwear?	Yes	No	
9.	Do you change your footwear regularly?	Yes	No	
10.	Do you go for a foot check-up regularly?	Yes	No	
	If yes, when?	Once a year	Only during illness	
Total score				

Appendix B

Michigan Neuropathy Screening Instrument

Part A

·      The MNSI questionnaire is self-administered. Responses are added to obtain a total score.

·      Yes responses to questions 1-3, 5-6, 8-9, 11-12, 14-15 are each counted as 1 point.

·      No responses to questions 7 and 13 each count as 1 point. Question 4 was considered to be a measure of impaired circulation.

·      Question 10, a measure of general asthenia, was not included in the published scoring algorithm.

·      A score of ≥ 7 will be considered abnormal.

Part B

During the MNSI B examination, a health professional inspects:

·      Each foot for deformities, dry skin, calluses, infections, fissures, or any other abnormality - score of 1.

·      Each foot is inspected for ulcers, and each foot with an ulcer is scored as 1.

·      The ankle reflexes are elicited

a) If present, scored as 0

b) If absent, scored as 1

Jendrassik maneuver is performed now

a) If present, scored as 0.5

b) If absent, scored as 1

§ Vibration sensation in the great toe using a 128-Hz tuning fork. If the examiner senses the vibration on his or her finger for

a) <10 seconds longer than the subject feels it in the great toe -PRESENT

b) ≥10 seconds (scored as 0.5) - decreased

c) Absent (scored as 1)

The total possible score is 10 points, and a score ≥ 2.5 is considered abnormal.

10-g monofilament is used for monofilament test.
